# Circulating CTRP1 Levels Are Increased and Associated with the STOD in Essential Hypertension in Chinese Patients

**DOI:** 10.1155/2019/4183781

**Published:** 2019-06-02

**Authors:** Zhengjia Su, Shuya Tian, Wei Liang

**Affiliations:** Department of Geratology, Ruijin Hospital, Shanghai Jiao Tong University School of Medicine, Shanghai 200025, China

## Abstract

This study aimed to investigate the correlation between complement C1q tumor necrosis factor-related protein 1 (CTRP1) and subclinical target organ damage (STOD) in essential hypertension (EH). 720 patients were enrolled in this study, including 360 healthy subjects and 360 patients with EH. The EH group included 183 patients complicated with STOD and 177 patients without STOD. In the STOD group, there were 87 patients with left ventricular hypertrophy (LVH), 32 patients with microalbuminuria (MAU), and 58 patients with complication of LVH and MAU. Enzyme-linked immunosorbent assay (ELISA) was used to detect the CTRP1, adiponectin (APN), interleukin-6 (IL-6), and tumor necrosis factor-*α* (TNF-*α*). We found that CTRP1 levels were higher in patients with EH than those in healthy subjects; moreover, the level of CTRP1 of patients in the group complicated with EH and STOD was increased compared with EH patients without STOD. CTRP1 levels in the group complicated with LVH and MAU were significantly higher than those in the LVH group and the MAU group. Furthermore, APN, CTRP1, and IL-6 were three factors that influenced the STOD of EH patients, among which CTRP1 and IL6 were positively related with the complication of hypertension and STOD. In conclusion, CTRP1 levels are increased and associated with the STOD (heart and kidney) in essential hypertension, which can be regarded as a novel biomarker in the prediction of prognosis for patients with essential hypertension.

## 1. Introduction

Hypertension accounts for the largest amount of attributable cardiovascular (CV) mortality in the world. STOD is a prognostic marker for future cardiovascular events [1], while multiorgan STOD carries a greater risk compared with single STOD [2, 3]. In addition, evidence of STOD may help to make the choice of the appropriate therapeutic pharmacological strategy in hypertensive patients. To this purpose, biomarkers are increasingly being used to assess STOD at increasingly early stage.

It is recognized that inflammatory adipokines including TNF-*α* can influence vasocontractility and regulate blood pressure [4]. An adiponectin family paralog, CTRP, has recently been identified. High levels of CTRP1 are positively correlated with metabolic syndrome, adiponectin deficiency, platelet aggregation, and hypertension [5, 6]. CTRP1 levels are increasing in patients with coronary artery and heart disease [7–9]. In addition, hypertensive patients also have increased CTRP1 levels, and CTRP1 stimulates aldosterone production via upregulation of the transcription of cytochrome P450 11*β*-hydroxylase 2 (Cyp11b2), which is the rate-limiting enzyme for aldosterone production [10]. These studies support the view that CTRP1 plays an important role in the regulation of cardiovascular function. However, the relationship between CTRP1 and STOD in essential hypertension remains unknown.

In the present study, we enrolled patients with essential hypertension and assessed the presence of heart and kidney damage. Here we investigated whether CTRP1 levels were associated with the STOD in essential hypertension.

### 1.1. Subject and Method

#### 1.1.1. Subject

Following the guidelines established by the European Society of Cardiology (ESC) and the European Society of Hypertension (ESH) in 2018, essential hypertension patients were defined as individuals whose two consecutive measurements of systolic blood pressure (SBP) were ≥140 mmHg and/or diastolic blood pressure (DBP) ≥90 mmHg [11]. All subjects had not other clinical complications as diabetes, hepatic inadequacy, congestive cardiac failure, coronary heart disease, valvular heart disease, cardiomyopathy, arrhythmia, autoimmune disease, apoplexy and cerebral infarction, and target organ damage. We regarded STOD as patients with LVH and/or MAU in this study.

A total of 720 subjects, comprising 360 patients with EH and 360 healthy subjects, were recruited at Geriatric Department of Ruijin Hospital affiliated to Shanghai Jiao Tong University School of Medicine during December, 2015 to November, 2017.

Patients diagnosed with EH were divided into two groups in accordance with the complication of STOD: 177 patients complicated with STOD and 183 patients without STOD. In the STOD group, 58 patients had complication of LVH and MAU, 32 patients had MAU, and 87 patients had LVH.

#### 1.1.2. Assessment and Definition

*(1) Definition of Left Ventricular Hypertrophy*. American HPSONOS5550 ultrasound machine was applied, with 2.5 MHz probe. Echocardiograms were obtained at rest with patients' supine in the left lateral position, using standard parasternal and apical views. Left ventricular mass (LVM) was derived using the formula described by Devereux et al. [12]: LVM(g)=0.80 × 1.04[(VSTd+LVIDd+PWTd)^3^–(LVIDd)^3^]+0.6, where VSTd is ventricular septal thickness at end diastole, LVIDd is left ventricular internal dimension at end diastole, and PWTd is left ventricular posterior wall thickness at end diastole.

Left ventricular mass was indexed for body surface area (BSA). The presence of LVH was defined as left ventricular mass index (LVMI) more than 115 g/m^2^ in men and more than 95 g/m^2^ in women in accordance with the definition of 2018 ESC/ESH Guidelines for the management of arterial hypertension [11].

*(2) Definition of Microalbuminuria*. A random urine sample was collected during the first morning void. Microalbuminuria was defined as a urinary albumin-to-creatinine ratio (UACR) of 2.5 to 25 mg/mmol in males and 3.5 to 35 mg/mmol in females, in accordance with the definition of the National Clinical Guideline Centre (UK) [13].

MAU reflects the prophase renal damage of hypertension [14]. Value of urinary albumin/creatinine (UACR) was regarded as test way of MAU. The first morning urine was collected and detected: UACR ≥2.5mg/mmol (male) and ≥3.5mg/mmol (female) was regarded as MAU.

#### 1.1.3. Blood Samples and ELISA

Blood samples were centrifuged at 3, 500 rpm at 4°C for 15 min. The plasma was stored at −80°C until further use. The concentrations of CTPR1, APN, IL-6, and TNF-*α* were detected using ELISA kit (Shanghai Senxiong Bio-Tech CO. Ltd.) following the manufacturer's instructions.

#### 1.1.4. Statistical Treatment

x ± s was used to stand for the measurement data with normal distribution; frequency (n) or percentage (%) was used to represent the enumeration data. T-test or variance analysis was used for measurement data; *χ*2 test or rank test was used for enumerable data; linear correlation regression analysis was used for interrelationship of different parameters; Pearson linear correlation regression analysis was used for correlation test. P<0.05 is regarded as statistically significant difference.

## 2. Results

### 2.1. Characteristics of Study Subjects

Characteristics of subjects with EH (n = 360) and the corresponding age- and sex-matched controls (n = 360) were described in Table 1. Compared with controls, the EH patients had greater values of BMI, FGLU, TG, LDL-C, CRP, LVMI, UACR, CTRP1, TNF-*α*, and IL-6. Levels of HDL-C and APN decreased significantly in EH group (p<0.05 for all parameters) (Table 1).

The EH patients were then divided into two groups in accordance with the complication of STOD. We identified EH patients with STOD as hypertension group 1 and EH patients without STOD as hypertension group 2.

The characteristics of group 1 (n=177) and the corresponding age- and sex-matched group 2 (n=183) are described in Table 1. Compared with group 2, the patients in group 1 had greater values of LVMI and UACR (P>0.05 for all parameters). Two groups had similar levels of SBP, DBP, BMI, FGLU, TC, TG, LDL, CRP, and HDL (P>0.05 for all parameters) (Table 2).

### 2.2. CTRP1 Levels Were Increased in EH Patients with STOD

**Figure 1:**
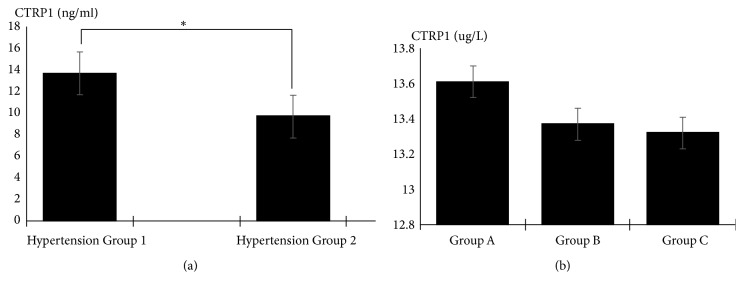
(a) Comparison of CTRP1 concentration between group 1 and group 2. *∗*P<0.05. (b) Comparison of CTRP1 concentration among three subgroups. CTRP1 levels elevated in Group A significantly.

The plasma CTRP1 levels were greatly increased in group 1 compared with group 2 (13.73±2.67 ng/mL versus 9.73±1.99 ng/mL, p=0.001, Figure 1(a)). We additionally subdivided the patients in group 1 into 3 groups of LVH complicated with MAU group (Group A), MAU group (Group B), and LVH group (Group C). As the number of target organ increased, the plasma CTRP1 levels elevated in Group A significantly (13.61±2.61 ng/mL,13.37±2.45 ng/mL, 13.32±2.69 ng/mL, Figure 1(b)).

**Figure 2:**
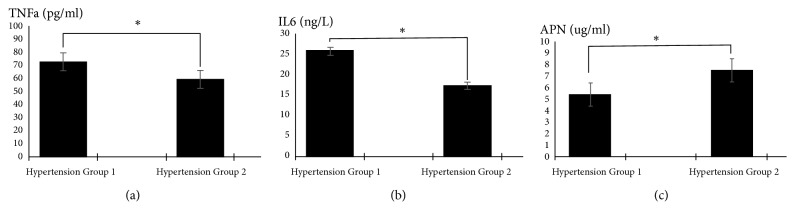
(a) Comparison of TNF-*α* concentration between group 1 and group 2. *∗*P<0.05. (b) Comparison of IL-6 concentration between group 1 and group 2. *∗*P<0.05. (c) Comparison of APN concentration between group 1 and group 2. *∗*P<0.05.

In addition, other inflammatory adipokines including APN, TNF-*α*, and IL-6 were analyzed in these groups. TNF-*α* and IL-6 levels were significantly increased in group 1 (P<0.05) while APN levels were significantly decreased (73.38±25.60 pg/mL versus 59.72±17.34 pg/mL, 25.90±5.02 ng/L versus 17.31±2.15 ng/L, 5.38±0.87 ug/mL versus 7.46±1.63 ug/mL, p=0.001, Figure 2).

**Figure 3:**
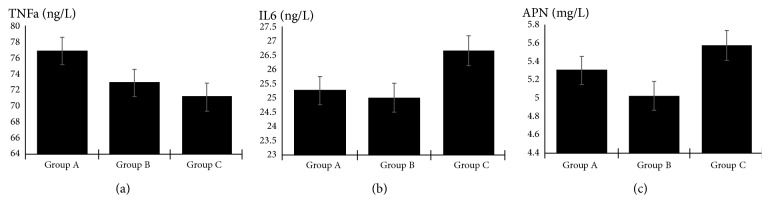
(a) Comparison of TNF-*α* concentration among three subgroups. (b) Comparison of IL-6 concentration among three subgroups. (c) Comparison of APN concentration among three subgroups.

In the subgroup analysis stratified by STOD, APN levels decreased significantly in group A; however, the plasma concentrations of TNF-*α* and IL-6 were not significantly different among the 3 subgroups (Figure 3, Table 3).

### 2.3. CTRP1 Increased the Risk of STOD in EH Patients

We applied the multivariable logistic regression to adjust for BMI, TG, LDL, CRP, and HDL, which were verified to be associated with hypertension, although there were no significant differences of these covariates in group 1 and group 2. A higher CTRP1 level had an increased risk for the STOD in EH (OR range 1.687-3.325, p=0.001). The risk of STOD also elevated as the IL-6 level increased (OR range 1.615-2.728, p=0.001). There also was an association between APN and STOD risk (Table 4).

In addition, when the STOD group stratified into three subgroups, we could still find the relation between CTRP1, as well as APN, and organ damage risk, respectively, in 3 groups. The correlation between IL-6 and the organ damage risk was only in LVH group (Table 5).

### 2.4. Analysis for the Association among CTRP1, APN, TNF-*α*, and IL-6

**Figure 4:**
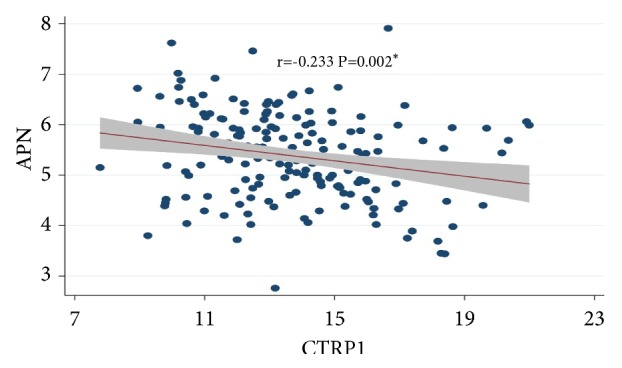
Scatter diagram of correlation between CTRP1 and APN. Note: CTRP1 (r value was -0.233) was significantly negatively correlated with APN (*∗*P<0.05) in the hypertension group 1 and the “shade” represents 95%CI of fitted line.

As CTRP1, TNF-*α* and IL-6 levels were found to increase in STOD, while APN levels decreased, the association among these inflammatory adipokines was further investigated. Pearson analysis showed that CTRP1 was negatively correlated with APN (P<0.05), while CTRP1 levels were not significantly associated with TNF-*α* and IL-6 (Figure 4, Table 6).

## 3. Discussion

A thorough evaluation of STOD, an independent determinant of CV risk, has become a key step in the initial management of patients with essential hypertension. Biomarkers are being increasingly used for the assessment of STOD which has relevant impact on therapeutic strategies.

In this study, we demonstrated that CTRP1 levels were increased in EH patients compared with healthy subjects and the CTRP1 levels in the hypertensive patients with STOD were significantly higher than those in the patients without STOD. We further observed that the CTRP1 levels were elevated according to the severity of STOD. The CTRP1 levels in two-organ damage group including LVH and MAU were higher than those in single-organ damage group, whereas the CTRP1 levels were not markedly different between LVH group and MAU group. This study also indicated that CTRP1 might be the risk of STOD in essential hypertension. Moreover, we found that CTRP1 was negatively correlated with APN.

Some previous reports have suggested that the CTRP1 levels were increased in patients with stable coronary heart disease and congestive heart failure [7, 15]. It has also been shown that CTRP1 levels are increased in hypertensive patients [5]. In agreement with these findings, our study revealed that the plasma CTRP1 levels were increased in the EH patients, furthermore, they were increased significantly in EH patients with STOD including MAU and/or LVH, regardless of pharmacologic treatment of hypertension.

It was likely that the secretion of CTRP1 was stimulated by the overall inflammatory status in hypertension [16, 17]. So we also detected other inflammatory adipokines including APN, TNF-*α*, and IL-6 in the study. We found that TNF-*α* and IL-6 levels increased in patients with STOD, however, whether these two inflammatory cytokines were associated with the severity of STOD kept uncertain. Some studies have demonstrated that [18] CTRP1 was related with the chronic inflammation and participated in the signal pathway that activates the AMPK, AKT, and P42/44 MAPK. Jun-nan Tang et al. [19] discovered that IL-6 secreted by vascular smooth muscle cell in hypertension patients could promote the increase of CTRP1, suggesting that CTRP1 may act as the proinflammatory mediator to amplify the inflammation of vessel cells. In animal studies, the proinflammatory mediators including TNF-*α* are involved in the induction of CTRP1 expression in adipose tissue [20]. A previous study reported by Ying Yang [7] showed that the IL-6 mRNA level was elevated by a treatment with CTRP1. However, our data showed that no significant correlation was observed between CTRP1 either TNF-*α* or IL-6. Thus, future clinical studies will be needed to clarify the relationship of inflammatory factors with CTRP1 and the severity of STOD in a larger population.

APN, which is another important circulating adipocytokine, shares multiple common biochemical features with CTRPs and has a similar globular head and trimetric basic protein structure [21]. It was reported that APN deficient mice exhibited increased levels of CTRP1 compared with control mice, indicating the negative association between adiponectin and CTRP1 levels [22]. It also demonstrated [16, 23] the negative association between CTRP1 and APN in diabetes rats and in diabetes. In the present study, Pearson correlation analysis demonstrated that CTRP1 was negatively correlated with APN, which was consistent with the previous report. Anti-inflammatory and antiatherogenic mechanism of APN may include eNOS activating through PI-3K pathway, which can promote NO and lead to the endothelium-dependent vasodilation, inhibiting the formation of white cell colony and the secretion of TNF-*α*. The anti-inflammatory mechanism may also be mediated by AMPK-mediated regulation of NF-KB and Akt kinase B [24, 25].

Several limitations of the present study should be considered. The pharmacologic treatment in this study was not given, which may have confounded the association between the CTRP1 and STOD. Due to the restriction of the number of damaged organs, we did not investigate the CTRP1 levels in patients with more than two target organs, which should be further studied.

## 4. Conclusion

Our study demonstrated that the levels of CTRP1 in the plasma were higher in the essential hypertensive patients with STOD. CTRP1 might be the risk of STOD in essential hypertension, which was also associated with the severity of STOD. Moreover, we found that CTRP1 was negatively correlated with APN. CTRP1 could be regarded as a novel biomarker in the prediction of prognosis for patients with essential hypertension. However, further studies are necessary to explain the precise role of CTRP1 in EH patients with STOD.

## Figures and Tables

**Table 1:** Clinical and biochemical characteristics of hypertension group and control group (x±s).

Indicator	Hypertension group (n=360)	Control Group (n=360)	*χ* ^2^/t value	P value
Age	58.27±15.08	58.91±13.16	-0.600	0.548
Gender			0.358	0.549
Female	168(46.7)	160(44.4)		
Male	192(53.3)	200(55.6)		
SBP(mmHg)	150.29±9.83	116.73±10.56	44.117	0.001
DBP(mmHg)	79.97±11.42	70.00±7.06	14.090	0.001
BMI(kg/m2)	24.47±3.38	23.82±2.42	2.978	0.003
FGLU(mmol/L)	5.49±0.93	5.36±0.80	2.043	0.041
FIN(mIU/L)	11.48±1.78	11.50±1.88	-0.156	0.876
HOMA	1.96±0.58	1.93±0.41	0.857	0.392
TC(mmol/L)	4.74±1.03	4.63±1.19	1.250	0.212
TG(mmol/L)	1.85±0.59	1.61±0.44	6.108	0.001
LDL(mmol/L)	3.20±0.84	2.79±0.81	6.617	0.001
HDL(mmol/L)	1.09±0.27	1.24±0.33	-6.702	0.001
CRP(mg/L)	7.20±2.14	6.16±1.31	7.850	0.001
LVMI(gym2)	116.82±18.30	83.71±24.83	20.365	0.001
UACR(mg/mmol)	2.25±0.64	1.53±0.47	17.227	0.001
APN(mg/L)	6.44±1.67	9.91±1.97	-25.554	0.001
CTRP1(ug/L)	11.70±3.09	8.22±1.41	19.456	0.001
TNFa(ng/L)	66.44±22.81	35.24±3.75	25.609	0.001
IL6(ng/L)	21.54±5.76	14.34±2.02	22.356	0.001

**Table 2:** Clinical and biochemical characteristics of hypertension group 1 and hypertension group 2 (x±s).

Indicator	hypertension group 1 (n=177)	hypertension group 2 (n=183)	*χ* ^2^/t value	P value
Age	58.25±15.07	58.75±13.29	-0.226	0.822
Gender			0.016	0.899
Female	82(46.3)	86(47.0)		
Male	95(53.7)	97(53.0)		
SBP(mmHg)	150.41±10.00	150.16±9.69	0.239	0.811
DBP(mmHg)	80.86±11.57	79.11±11.23	1.451	0.148
BMI(kg/m2)	24.31±3.37	24.62±3.39	-0.886	0.376
FGLU(mmol/L)	5.56±0.93	5.42±0.93	1.508	0.132
FIN(mIU/L)	11.45±1.71	11.50±1.85	-0.291	0.771
HOMA	1.93±0.55	1.99±0.60	-1.030	0.304
TC (mmol/L)	4.77±1.05	4.71±1.01	0.487	0.627
TG(mmol/L)	1.87±0.58	1.82±0.60	0.726	0.468
LDL(mmol/L)	3.17±0.84	3.23±0.85	-0.588	0.557
HDL(mmol/L)	1.09±0.27	1.09±0.28	0.155	0.877
CRP(mg/L)	7.26±2.17	7.15±2.11	0.463	0.644
LVMI(gym2)	128.90±16.11	105.13±11.41	16.191	0.001
UACR(mg/mmol)	2.42±0.66	2.09±0.58	5.056	0.001

**Table 3:** Characteristics among three subgroups (x±s).

Indicator	Group A n=58	Group B n=32	Group C n=87	F	P
APN (mg/L)	5.32±0.86	5.02±0.98	5.60±0.79	5.512	0.005
CTRP1 (ug/L)	13.61±2.42	13.37±2.45	13.32±2.70	7.609	0.001
TNF-*α* (ng/L)	76.84±20	72.86±29.05	71.12±27.42	0.86	0.425
IL-6 (ng/L)	25.23±4.93	24.99±5.31	26.65±4.99	1.933	0.148
LVMI (gym2)	132.73±12.83	103.41±11.48	135.58±8.66	108.907	0.001
UACR (mg/mmol)	2.90±0.29	3.07±0.36	1.84±0.42	196.457	0.001

Group A: EH patients with both LVH and MAU.

Group B: EH patients with MAU.

Group C: EH patients with LVH.

**Table 4:** Multiple logistic regression analysis of factors influencing STOD in hypertension.

Indicator	B	S.E.	Wald	df	P	OR (95%CI)
APN	-1.182	0.250	22.317	1	0.001	0.307 (0.188, 0.501)
CTRP1	0.862	0.173	24.831	1	0.001	2.368 (1.687, 3.325)
TNF-*α*	0.022	0.014	2.249	1	0.134	1.022 (0.993, 1.051)
IL-6	0.741	0.134	30.692	1	0.001	2.099 (1.615, 2.728)
BMI	0.034	0.088	0.151	1	0.698	1.035 (0.870, 1.231)
TG	0.183	0.554	0.109	1	0.741	1.201 (0.405, 3.560)
LDL	-0.671	0.354	3.600	1	0.058	0.511 (0.255, 1.022)
HDL	-1.839	1.097	2.808	1	0.094	0.159 (0.019, 1.366)
CRP	0.007	0.149	0.002	1	0.964	1.007 (0.752, 1.348)
Constant	-15.913	4.452	12.778	1	0.000	-

**Table 5:** Multiple logistic regression analysis in subgroups.

	Group A		Group B		Group C	
	OR (95%CI)	P	OR (95%CI)	P	OR (95%CI)	P
APN	0.487 (0.349,0.680)	0.001	0.503 (0.317,0.798)	0.004	0.664 (0.530,0.833)	0.001
CTRP1	1.377 (1.214,1.561)	0.001	1.121 (0.949,1.324)	0.018	1.058 (0.953,1.174)	0.024
TNF-*α*	1.007 (0.994,1.021)	0.292	1.023 (1.001,1.046)	0.039	1.012 (0.999,1.025)	0.061
IL6	1.010 (0.950,1.074)	0.746	0.979 (0.894,1.072)	0.642	1.232 (1.157,1.312)	0.001

Group A: EH patients with both LVH and MAU.

Group B: EH patients with MAU.

Group C: EH patients with LVH.

**Table 6:** Pearson analysis of the association among CTRP1, APN, TNF-*α*, and IL-6.

	APN		CTRP1		TNFa		IL6	
	r	P	r	P	r	P	r	P
APN	-	-	-0.233	0.002	-0.018	0.813	-0.082	0.280
CTRP1	-0.233	0.002	-	-	-0.017	0.825	0.037	0.624
TNFa	-0.018	0.813	-0.017	0.825	-	-	0.064	0.395
IL6	-0.082	0.280	0.037	0.624	0.064	0.395	-	-

## Data Availability

The data used to support the findings of this study are available from the corresponding author upon request.
